# Editorial: Exercise prescription in metabolic diseases: An efficient medicine towards prevention and cure

**DOI:** 10.3389/fphys.2022.947365

**Published:** 2022-07-22

**Authors:** Georges Jabbour, Shahrad Taheri

**Affiliations:** ^1^ Department of Physical Education, College of Education, Qatar University, Doha, Qatar; ^2^ National Obesity Treatment Centre, Qatar Metabolic Institute, Hamad Medical Corporation, Doha, Qatar; ^3^ Weill Cornell Medicine Qatar, Ar-Rayyan, Qatar; ^4^ Weill Cornell Medicine, New York, NY, United States

**Keywords:** physical activity, exercise, exercise prescription, metabolic diseases, moderate exercie, high intensity exercise

Metabolic disease encompasses excess adiposity, dysglycaemia, type 2 diabetes mellitus, dyslipidaemia, non-alcoholic fatty liver disease (NAFLD), and hypertension that translate to excess cardiovascular disease and increased mortality. The prevalence of metabolic disease is increasing in prevalence, driven by excessive intake of energy dense food and physical inactivity, and is a major challenge for healthcare services. Physical inactivity is a major contributor to the development of metabolic disease and regular exercise can prevent, delay, and control metabolic disease.

Based on extensive evidence of health benefits, physical activity is widely recommended today for individuals with or at high risk of developing metabolic disease. Furthermore, exercise prescription, particularly aerobic exercise, has been used as an efficient approach to counteract many metabolic impairments including type 2 diabetes (Jabbour and Iancu, 2017a). Undertaking the recommended levels of physical activity, however, is challenging and there are several individual and environmental barriers to achieving and maintaining the recommendations. Because of this, recently there is great interest in very brief high-intensity exercise in the form of interval training in shorter laps of time which has demonstrated potential health benefits, appears to be highly motivating and tolerable for participants (Whyte et al., 2010; Jabbour et al., 2017b; Jabbour and Iancu, 2017c). According to Whyte et al. (2010), very high-intensity sprint interval training improved several metabolic risk factors such as an increase in resting fat oxidation rate in the fasted state and a decrease in resting carbohydrate oxidation in the fasted state compared with baseline. For Parimbelli et al., training based on short-duration high-intensity exercise improves overall metabolism and aerobic fitness in a case of carnitine palmitoyltransferase II deficiency. Also, Jabbour et al. (2017b), Jabbour et al. (2015) reported significant improvements of many physiological and metabolic parameters immediately and not exclusively at post intervention at 2 weeks. Lipid oxidation improved among obese participants in this study despite no change in body weight. Gao et al. reported in their first meta-analysis that focus on effect of long-term exercise on liver lipid metabolism in Chinese patients with. NAFLD (also called MAFLD for metabolic associated fatty liver disease) is a relatively new addition to the metabolic disease cluster. NAFLD is not only associated with liver manifestations such as steatohepatitis, cirrhosis, and hepatocellular carcinoma, but is also associated with excess cardiovascular mortality.

In an older population, Park et al. reported that above moderate-active physical activity (PA) levels were associated with lower body weight, body fat mass, percent body fat and higher free-fat mass and higher HDL-C than low-active PA. Also, above high-active PA levels were associated with lower cardiometabolic risk factors, waist circumference, total cholesterol, and triglycerides than low- and moderate-active PA. In terms of the risk of osteoporosis, sarcopenia, obesity, sarcopenic obesity, and cardiac metabolic risk factors according to PA levels, the prevalence of cardiometabolic disease was significantly lower in high-active PA (vs. low- and moderate-active); waist circumference, and HDL-C were significantly better in moderate- and high-active PA (vs. low-active), respectively, and triglycerides significantly better in high-active PA (vs. low- and moderate-active). Today, it still too much to do before being able to clarify all mechanisms involved in such improvement. A recent study of Luo et al. concluded that an exercise training accompanied by dietary fat regulation modulate TRIB3 and macrophage phenotype to attenuate insulin resistance. This latter, is widely viewed as a common basis of the etiology of metabolism diseases related to obesity (Katula et al., 2013).

To summarize, current evidence suggest that long-term exercise and high intensity exercise can improve several key health indicators. Concurrent with the new evidence, establishing an updated “consensus of PA/exercise intervention” (e.g., modality; individualization) become necessary. An accurate exercise prescription (moderate exercise/intensity vs. vigorous exercise/intensity) is still confusing to enable the determination of the exercise mode. In fact, the common practice of prescribing exercise at a fixed metabolic rate (# of METs) or percentage of maximal heart rate or of maximal oxygen uptake (V̇O2max) does not acknowledge the individual variability of these metabolic boundaries. As training adaptations occur, these boundaries will change in absolute and relative terms (MacIntosh et al.). MacIntosh et al. provide a framework for understanding “moderate to vigorous” physical activity intensities and advanced strategies in terms of individual identification for exercise prescription. The authors recommend that expressing the exercise according to ventilatory threshold 1 and 2 (VT1 and VT2) or lactate threshold 1 and 2 (LT1 and LT2) is most useful for accurate exercise prescription.

Despite studies reporting numerous benefits of exercise for both the prevention and the treatment of metabolic disease, large variability in the inter-individual changes and improvement at post-intervention has been reported. It is important to highlight that one of the major challenges were in controlling for factors that differ between participants as well as in exercise prescription. Other issues are small sample sizes and lack of long-term follow-up to assess the impact of the exercise intervention. Prescribing exercise is promising for both prevention and reversal of metabolic disease. The challenge will be to incorporate this into daily lives to ensure that potential long-term benefits are realized.
